# Mandrills learn two-day time intervals in a naturalistic foraging situation

**DOI:** 10.1007/s10071-020-01451-7

**Published:** 2020-11-30

**Authors:** Kavel C. D. Ozturk, Martijn Egas, Karline R. L. Janmaat

**Affiliations:** 1 Department of Evolutionary and Population Biology, Institute of Biodiversity and Ecosystem Dynamics, University of Amsterdam, Science Park 904, 1098 XH Amsterdam, The Netherlands; 2 Department of Cognitive Psychology, Leiden University, Pieter de la Court, Wassenaarseweg 52, 2333 AK Leiden, The Netherlands; 3 ARTIS Amsterdam Royal Zoo, Plantage Kerklaan 38-40, 1018 CZ Amsterdam, The Netherlands

**Keywords:** Time estimation, Interval learning, Temporal memory, Synchrony cues, Foraging cognition, Mandrills

## Abstract

Primates display high efficiency in finding food in complex environments. Knowledge that many plant species produce fruit simultaneously, can help primates to anticipate fruit finding at the start of fruiting seasons. Knowledge of elapsed time can help primates decide when to revisit food trees to find ripened fruit and to return before competitors find these fruits. To investigate whether mandrills are able to learn time intervals of recurring food, we recorded the foraging choices of captive mandrills in a group setting. We used a procedure with renewable food rewards that could be searched for: carrots and grapes, hidden underground in specific places with different renewal intervals (2 and 5 days, respectively). We monitored the first choice of location for individuals, if other individuals had not already searched at the same location, to exclude possible effects of individuals following others rather than relying on memory. Throughout the study, the mandrills became increasingly likely to first search at carrot locations on carrot days, while the probability of them searching at carrot locations decreased on days without carrot. Due to model instability, our results were inconclusive about an effect of grape days on the choice of the mandrills. Cues provided by conspecifics indicating the availability of simultaneously emerging food rewards did not affect the choice of the mandrills. We conclude that mandrills can take into account elapsed time in a foraging context. Thereby, this study indicates how mandrills can use temporal cognitive abilities to overcome temporal challenges of food-finding in a group setting.

**Supplementary Information:**

The online version contains supplementary material available at 10.1007/s10071-020-01451-7.

## Introduction

Acquiring food is essential for the survival of all animals, including primates. Primates, however, have relatively large brains that are energetically expensive and require a large proportion of their energy budget, i.e. between 9 and 12% (Mink et al. 1981), placing a premium on those individuals that are better at acquisition than others. Most wild primates need to sustain these energetic costs in highly competitive environments with high variability in food availability both in space and time (Gautier-Hion 1990; Chapman et al. 2005; Houle et al. 2006; Van Woerden et al. 2012). Hence, the question arises as to how primates are able to find and obtain food in such challenging environments.

It has been suggested that wild primates, to successfully acquire food, are reliant on a variety of cognitive abilities (Zuberbühler and Janmaat 2010; Janmaat et al. 2016; Trapanese et al. 2018). For example, studies suggest that primates and other animals that strongly rely on sessile food sources benefit from remembering *where* specific types of food are located to successfully forage (Milton 1981, 2000; Janmaat and Chancellor 2010; González-Gómez et al. 2011; Fagan et al. 2013). Another important cognitive ability that may allow primates to locate food is the ability to use temporal knowledge. With the use of such abilities, primates could be able to temporally locate food with seasonal and annual fluctuation patterns in the wild (Milton 1981, 2000; Trapanese et al. 2018; Janmaat et al. 2016). Furthermore, keeping track of elapsed time might provide primates with the knowledge when to return to a specific tree that had unripe fruits during the last visitation. However, only a limited number of studies have provided evidence that primates can remember elapsed time since a specific event. These studies have indicated that chimpanzees (*Pan troglodytes*), bonobos (*Pan paniscus*), black capuchin monkeys (*Sapajus nigritus*), chacma baboons (*Papio ursinus*) and rhesus macaques (*Macaca mulatta*) can keep track of elapsed time (Hampton et al. 2005; Martin-Ordas et al. 2010; Noser and Byrne 2015; Janson 2016).

Most of these studies have focused on the capacity of primates to remember elapsed time intervals that ranged from several minutes to several hours (Hoffman et al. 2009; Martin-Ordas et al. 2010). Such short time intervals might not be very relevant in a foraging context. Remembering time intervals of several days may be more ecologically relevant, since knowledge about the number of days that have passed in combination with knowledge of fruit maturation rates—which can last days to several months (Tutin et al. 1996; Spironello 1999; Ratiarison and Forget 2011)—can enable animals to outcompete competitors in having first access to ripe fruit. To our knowledge, only three studies have been able to provide evidence that time intervals longer than a day can be remembered by primates (Noser and Byrne 2015; Janson 2016; Tujage and Janson 2017). These studies have shown that both capuchin monkeys and chacma baboons have such time-estimating capacities. However, information regarding this temporal cognitive capacity in other primate species is lacking (Trapanese et al. 2018; Zuberbuhler and Janmaat 2010).

Apart from temporal cognitive abilities, studies have indicated that some primates use additional, cognitive abilities to localize fruit when a species comes to season. Several studies have shown that some primates have the capacity to use positive cues (cues based on the occurrence) and negative cues (based on the non-occurrence) to infer the presence and absence of food, respectively (Heimbauer et al. 2012; Call 2004). Furthermore, studies have provided evidence that various primates in the wild make use of fruiting synchrony, using the emergence of fruit in one tree of a species as an indicator that other trees of the same species will also bear fruit, to localize newly emerged fruit-bearing trees in season. This may be based on both positive and negative cues (Menzel 1991; Janmaat et al. 2012, 2013). Most tropical fruit-bearing trees produce fruit synchronously (Chapman et al. 1999; van Schaik et al. 1993) and monitoring as well as using fruit synchrony is suggested to be an additional effective way to localize fruit (Menzel 1991; Janmaat et al. 2012).

Provisioning experiments with renewable food sources offer a good possibility to study the temporal cognitive abilities of primates (Janson 2016). Such provisioning studies could be performed both in captive and wild primates. However, provisioning experiments in the wild may be constrained by various factors, such as neophobia of the subjects, competing background stimuli, and drastic alterations of the subjects’ behaviour (Janson 2012; Sugiyama 2015). Furthermore, food provisioning in the wild can affect the health of primates as a result of increased infection risks and human–primate disease transmission (Tiddi et al. 2019; Dunay et al. 2018).

Provisioning experiments in captivity could both be performed in strictly controlled settings and in naturalistic foraging settings. Naturalistic foraging settings that have foraging challenges that are comparable to the ones experienced in the wild and in which the subjects forage in a social groups can have increased ecological validity and result in increased memory performances (Janmaat 2019; Cronin et al. 2017; Menzel and Juno 1985). In this study, we examined the foraging cognitive capacities of captive mandrills (*Mandrillus sphinx*) in a naturalistic foraging setting. More specifically, we aimed to investigate whether mandrills were able to learn time intervals and use synchrony cues to locate food in a competitive setting that was expected to trigger motivation to learn.

Mandrills in the wild are mostly reliant on ripe fruit as a food source and share their habitat with at least 12 other primates that are potential food competitors (Harrison 1988; Rogers et al. 1996). In these competitive tropical habitats, food availability can vary substantially. Especially primate species that rely mainly on ripe fruit can face severe food scarcities for several weeks (Janmaat et al. 2014; Chapman et al. 2005; Terborgh 1986). Therefore, having first access to ripe fruits may be essential for mandrill survival. The capacity to learn time intervals is expected to provide mandrills with the ability to plan when they will revisit a location with unripe fruit and thereby provide them with first access to ripe fruits.

A cognitive enrichment procedure, with renewable food resources, was used to test the time-estimating capacities of captive mandrills in a group foraging setting. In this procedure, two food rewards that differ in sugar content (carrots, *Daucus carota*: ± 5 g sugar 100 g^−1^ carrot (Alasalvar et al. 2001); red grape, *Vitis vinifera*: ± 17 g sugar 100 g^−1^ grape (Muñoz-Robredo et al. 2011)) were hidden (buried in the ground) with different renewal intervals (2 and 5 days), at three marked locations each (six in total), thereby simulating different regeneration intervals in natural food sources and stimulating natural foraging behaviour, such as digging (King 1986). For comparability, we chose similar renewal intervals to the time intervals used in a study by Fuhrer and Gygax (2017) in which the temporal cognitive performances of sows (*Sus scrofa*) were studied by hiding two different food rewards.

We hypothesized that the mandrills would be capable of learning both the short- and long-time intervals. Based on this, we predicted that as time passed, the first location that the mandrills would search for food would coincide with one of the locations at which food was hidden on that specific day. So on grape days, we expected the mandrills to first search at a grape location and on carrot days, we expected them to first search at a carrot location. We expected that on days without any food hidden, the mandrills would still be more likely to search at a grape location. These expectations were based on the knowledge that fruiting intervals have some level of variation in the wild (Wrangham et al. 1998). Hence, it would make sense to still monitor a location, especially when it is a sweet and energy-rich food source. Grapes were sweeter than carrots and likely more preferred (Remis 2006; Remis and Kerr 2002).

In addition, we hypothesized that mandrills would be able to use positive and negative synchrony cues. We predicted that an individual would be more likely to search at a carrot location if a conspecific had already found carrots at one of the locations and likewise that an individual would be more likely to search for grapes when another individual had already found grapes. We also predicted that if an individual would search at a location and would fail to find food, other individuals would use this as a cue of absence and would, therefore, search at a location with the other type of food.

## Methods

### Subjects and housing

For this study, a group of mandrills consisting of ten individuals was observed between October 2018 and April 2019. The group consisted of one adult male (> 9 years), five adult females (> 4 years), one sub-adult male (6–9 years), two juveniles (1–3 years) and an infant that was born during the study. All mandrills were born in captivity and none of the mandrills was trained before with a similar cognitive enrichment procedure.

**Fig. 1:**
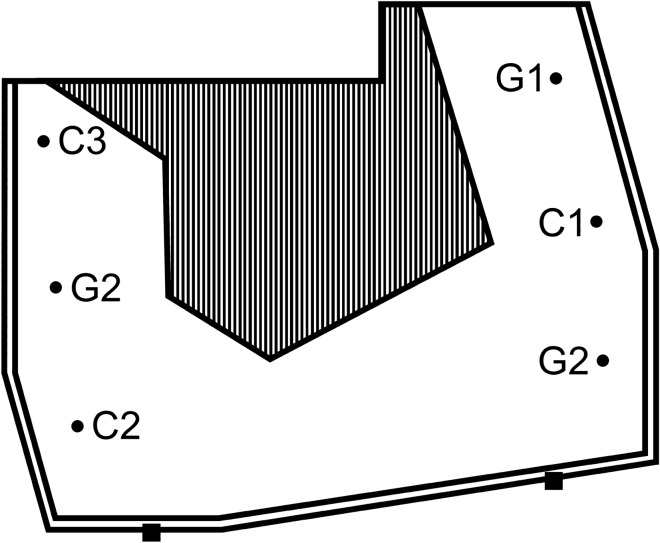
Schematic overview of the outside enclosure of mandrills and the food locations used in this study. The area with a striped pattern depicts a rock formation. *C* carrot location, *G* grape location. The numbers behind the letters represent the used numbering throughout the study. The black squares depict the placement of the two camera’s

The group was housed at Artis Royal Zoo in Amsterdam, The Netherlands, and had access to an inside and outside enclosure of 157 m^2^ (Fig. 1). A rock formation is located in the middle of the outside enclosure and the inside enclosure can be accessed through gates in this rock formation. Several tree trunks, which can be used for climbing, are attached to this rock formation. Additionally, the outside enclosure has permanent enrichments in the form of ropes and metal baskets and plastic balls, which are used as feeders. The bedding of the outside enclosure was composed of bark wood snippets and rocks.

Three times per day the mandrills were provided with vegetables, fruit, primate leaf-eater biscuits, monkey chow (Primacon) and muesli. Additionally, throughout the week, the mandrills received supplementary food, such as boiled eggs, crickets, and grasshoppers.

### Procedure

The trials for the study took place in the outside enclosure after it had been cleaned. They were usually conducted in the morning (between 08:30 and 11:00). During this time, the morning meal of the mandrills would be provided as well. All trials were monitored for one hour and were simultaneously recorded with two Nikkei action cameras (model number: Extreme X8S).

The study consisted of two phases. In the first *introductory phase*, the mandrills were introduced to the 6 food locations (three carrot locations and three grape locations), one at a time (Fig. 1). For this introductory phase, the same time intervals were used that would be used throughout the rest of the study (Table 1). Each location was introduced with two introduction days before the next location was added. We used this procedure to optimize the consolidation of memory as sleep is thought to contribute to its consolidation (Martin-Ordas and Call 2011). In the first introduction day, the food source would be buried in the ground and would additionally be placed on top of the soil at this location. From this first introduction day on, a willow branch was placed at this food location every morning, independent of whether food was buried or not. The second introduction day the food source was again buried at the location with the willow branch; however, no food was placed on top of the soil. Long willow branches (± 1.75 m) were placed at the grape locations and small willow branches (± 1 m) at the carrot locations. The burial of the food sources underground limited possible olfactory cues. The branches simulated two plants that were associated with the two underground food items from the corresponding species. The willow branch was placed to help the mandrills remember the food locations and to simulate a natural foraging situation as best as possible since mandrills in the wild also dig for roots and since closely related primate species, such as mangabeys (*Cercocebus atys*) are known to remove tree saplings and dig for associated seeds underground in the wild (King 1986; Unpublished data Janmaat). The abovementioned procedure resulted in an *introduction phase* that consisted of 6 days on which carrots were placed and eight days on which grapes were placed (during the introduction of the carrot locations the grapes were continued to be placed every fifth day) over a period of 37 days (i.e. 37 training trials; Table 1).

**Table 1:** Provisioning schedule of the introduction phase and the first 7 days of the testing phase

Day	Phase	Grape locations	Carrot locations
1	Introductory phase	Introduction grape location 1 (G1). Grapes at G1 placed visibly	
2	Introductory phase		
3	Introductory phase		
4	Introductory phase		
5	Introductory phase		
6	Introductory phase	Grapes at G1	
7	Introductory phase		
8	Introductory phase		
9	Introductory phase		
10	Introductory phase		
11	Introductory phase	Grapes at G1 and introduction G2. Grapes at G2 placed visibly	
12	Introductory phase		
13	Introductory phase		
14	Introductory phase		
15	Introductory phase		
16	Introductory phase	Grapes at G1 and G2	
17	Introductory phase		
18	Introductory phase		
19	Introductory phase		
20	Introductory phase		
21	Introductory phase	Grapes at G1 & G2 and introduction G3. Grapes at G3 placed visibly	
22	Introductory phase		
23	Introductory phase		
24	Introductory phase		
25	Introductory phase		
26	Introductory phase	Grapes at G1, G2 & G3	
27	Introductory phase		Introduction carrot location 1 (C1). Carrots at C1 placed visibly
28	Introductory phase		
29	Introductory phase		Carrots at C1
30	Introductory phase		
31	Introductory phase	Grapes at G1, G2 & G3	Carrots at C1 and introduction C2. Carrots at C2 placed visibly
32	Introductory phase		
33	Introductory phase		Carrots at C1 & C2
34	Introductory phase		
35	Introductory phase		Carrots at C1 & C2 and introduction C3. Carrots at C3 placed visibly
36	Introductory phase	Grapes at G1, G2 & G3	
37	Introductory phase		Carrots at C1, C2 & C3
38	Testing phase		
39	Testing phase		Carrots at C1, C2 & C3
40	Testing phase		
41	Testing phase	Grapes at G1, G2 & G3	Carrots at C1, C2 & C3
42	Testing phase		
43	Testing phase		Carrots at C1, C2 & C3
44	Testing phase		

From the introduction of each location on, a willow branch was placed at each specific location every morning, independent of whether there was food buried. Thus, from day 1 until the end of the study a willow branch was placed each morning at location G1. Similarly, from day 11 until the end of the study a willow branch was placed at location G2

During the second phase of the study, the *testing phase*, the burial of the food sources would continue with their respective time intervals, i.e. on every 5th day, grapes would be buried in the three respective grape locations and on every other day, carrots would be buried in the three respective carrot locations (see the video in Online Resource 2 for a trial during the *testing phase*). This resulted in four types of days during the testing phase: days without food buried, days with only carrots buried, days with only grapes buried and days with both types of food sources simultaneously buried. On days with grape, 500 g of red grape would be divided over the three grape locations and on days with carrot approximately 2, 5 winter-carrots (large Dutch domesticated carrots that roughly weigh 150 g per carrot) would be cut into smaller pieces and divided over the three carrot locations.

The carrots and grapes that the mandrills received normally as part of their diet, were taken out of their diet for the duration of this study and were provided solely in the enrichment procedure. Therefore, no carrots or grapes were provided other than those provided in the food locations during the trials of this study.

During the testing phase of the study, on February 27 on a carrot day, some parts of the outside enclosure had to be changed and during that day, the mandrills were not able to go into the outside enclosure. Therefore, this day and the following two days were omitted from the dataset and any further analysis.

### First choice and cue scoring

During the testing phase, we scored only the first choice of each individual, i.e. which location was visited and whether it was a grape or carrot location. With first choice, we mean that in the case that an individual went to multiple locations with grape and/or carrot, we only scored the first location that an individual visited. We considered that an individual ‘visited’ a location when the individual started removing the willow branch at the location or when it started to dig at the location in the cases that the individual did not remove the willow branch. Afterwards, this scoring was verified with the camera footage. We only used the choice of location for an individual, if another individual had not already searched for food at the same location within the same trial, to exclude possible effects of individuals following others rather than relying on their own memory and sense of time interval. For example, if one individual visited the first grape location and if subsequently, the first choice of another individual was the same location, only the first choice of the first individual was scored. The second individual would have no first choice that trial day since its first choice would not have been at an unvisited food location.

The camera footage was also used to score whether, at the time of the first choice of each individual, another individual had already searched for food at one of the six locations and thus had presented a potential cue. For this, we additionally scored what type of food location (carrot/grape) it was and whether there was food present at that location that day. These potential cues were scored as follows: cue grape: (1) *no*. (2) *yes, a cue of presence*. (3) *yes, a cue of absence*; cue carrot: (1) *no*. (2) *yes, a cue of presence*. (3) *yes, a cue of absence*. For instance, if the first choice of an individual at a grape location on grape day, was preceded by another individual finding grapes at another grape location, then cue grape would be scored as yes, a cue of presence.

### Statistical analysis: time-estimating and synchrony cues

To investigate the capacity of mandrills to learn the time intervals of carrots and grapes (i.e., whether the first choice of a subject would correspond with the type of food that was present that day) and to use synchrony cues, we used Generalized Linear Mixed Models (GLMM) of the lme4 package with a binomial error distribution (Bates et al. 2015). The binomial response variable was, for each mandrill, whether the individual searched at a grape location first or had not searched at a grape location (i.e. had searched at a carrot location as a first choice). This variable was composed of the first choice of every mandrill that had made a unique first choice—a first choice to an unvisited food location—that trial (i.e. when an individual had not made a unique first choice during a trial, then there was no data entry for that individual for that specific trial). For the statistical analysis, only the data from trials during the *testing phase* were used. Additionally, only data from days during which at least one individual had made a choice were included in the statistical analysis.

As fixed effects or main predictors, we included *cue grape* (three levels: no cue, cue of presence, cue of absence), *cue carrot* (three levels: no cue, cue of presence, cue of absence), *type of day* (four levels*: no food day, carrot day, grape day, both carrot and grape day*), *date* (as *days since start* of the testing phase). Because we expected the mandrills to learn over time, we also included an interaction between type of day and date. The identity of the subjects (*individual*) was used as a random effect. We included random slopes for all variables and the interaction between type of day and date within individual. To include random slopes for the categorical variables (*type of day*, *cue grape* and *cue carrot*) we manually dummy coded and centered them (Schielzeth 2010). Location was not used as a random effect since this would result in complete separation in which the various levels of location perfectly predict the response. Namely, the levels G1, G2, G3 of location always corresponded with the first choice of grape and the levels C1, C2, C3 corresponded always with carrot.

The number of unvisited grape and carrot locations varied throughout a trial due to individuals searching for food at the various locations, thereby limiting the available unvisited food locations for individuals that had yet to make a first choice. To control for this, we included the ratio of unvisited grape locations to the total unvisited locations as an offset term into the model.

Model stability was assessed by checking the effect of excluding the levels of the random effect individual one at a time, on the estimation of the coefficients. However, this revealed unstable estimates in the model in the interaction between type of day and days since start, due to a low number of first choices by some individuals within the levels of grape day and both carrot and grape day (see Table S1 in Online Resource 1 for the results of this model). Therefore, we constructed a second model.

#### Model 2: carrot day

In the second model, we removed the levels ‘grape day’ and ‘both carrot and grape day’ for the variable *type of day* (since the observations of choices on grape day were very low). In addition, we removed from the variable *cue grape* the level ‘cue of presence’—observations where a mandrill chose a location after another individual had given a cue that it was grape day. In other words, we removed all observations on grape days. This new model (*Model 2: carrot day*) allowed for the interpretation and estimation of the effect of the variable *type of day* with the remaining levels (*no food day, carrot day*), which was only possible after the removal of the levels *grape day* and *both carrot and grape day* which caused stability issues. *Model 2: carrot day* had as a binomial response whether subjects had searched at a carrot location first or a grape location (i.e., searching at a non-carrot location is equivalent to choosing a grape location as a first choice). The ratio of available carrot locations to the total available locations was included in the model as an offset term.

Model stability *Model 2: carrot day* was again assessed by excluding the levels of the random effect individual one at a time. This showed acceptable stability for the fixed effects (*cue carrots*, *type of day*, *date* and the interaction between *type of day* and *date*), with the exception of the fixed effect *cue grapes* (Table 2).

**Table 2:** Summary of results of binomial GLMM: effect of *type of day* on searching at a carrot location as a first choice (*N* = 194)

	Original Estimate	Std. error	*P-*value	Estimate min.^a^	Estimate max.^a^	Lower CI^b^	Upper CI^b^
(Intercept)	− 1.010	0.330		− 1.475	− 0.722	− 1.835	− 0.363
Cue grape (absence)	0.037	0.407	0.927	− 0.138	0.312	− 0.672	1.029
Cue carrot (absence)	0.633	0.550		0.235	0.944	− 0.608	1.663
Cue carrot (presence)	0.973	0.511		0.210	1.398	− 0.075	2.036
Cue carrot			0.275				
Days since start	− 0.641	0.233		− 0.792	− 0.543	− 1.188	− 0.199
Type of day (carrot)	0.732	0.417		0.270	1.360	− 0.052	1.622
Days since start*Type of day (carrot)	0.768	0.337	0.041*	0.569	1.257	0.119	1.511

Full model vs. control model comparison: *χ*^2^ = 9.026, df = 6, *p* = 0.029, *N*_first choice carrot_ = 83, *N*_individual_ = 5. Marginal *R*^2^ = 0.15

Significant *P*-values are marked with an asterisk

^a^Minimum and maximum estimated coefficients derived by taking out the levels of each random effect one at a time

^b^Bootstrapped 95% confidence interval

To test the effect of *type of day* and its interaction with *date* as a whole, the fit of this full model was compared to a null model with the use of a likelihood ratio test (Dobson and Barnett 2008). The null model in this instance lacked these two fixed effects, but other than that was identical to the full model.

#### Model 3: with days since grape

A third GLMM with a binomial error distribution was used, to analyse whether the mandrills were able to learn the carrot time interval, would not learn the exact grape-interval but would be more likely to search first at grape locations with increasing days since the last grape day (*Model 3: with days since grape*).

Here, the binomial response was whether a subject had searched at a grape location first or had not searched at a grape location. As fixed effects, we included *cue grape* (three levels: no cue, cue of presence, cue of absence), *cue carrot* (three levels: no cue, cue of presence, cue of absence), *carrot day* (two levels: yes, no), *days since grape* and *date* (as *days since start* of the testing phase) and two interactions between *carrot day* and *date* and *days since grape* and *date*. The identity of the subjects (*individual*) was used as a random effect. We included random slopes for all variables and their respective interaction within individual.

Originally *Model 3: with days since grape* included a three-way interaction between *date*, *days since grape* and *carrot day*, but the *p*-value for this interaction was higher than 0.05. The interpretation and estimation of the main effects are only possible after the removal of non-significant interaction terms. Hence, we omitted this interaction from the model and only included the interactions between *days since grape* and *date* and *carrot day* and *date*. The interaction between *days since grape* and *date* was still non-significant and, therefore, removed. The ratio of available grape locations to the total available locations was again included in the model as an offset term.

Model stability was assessed by excluding the levels of the random effect individual one at a time. This showed acceptable stability for *carrot day*, *date* and the interaction between *carrot day* and *date*. However, there was instability for the fixed effects of *days since grape*, *cues of grape* and *cues of carrot*.

To test the effects of days since grape, carrot day, and the interaction between *carrot day* and *date*, as a whole, the fit of this full model was compared to a null model with the use of a likelihood ratio test (Dobson and Barnett 2008). In this instance, the null model lacked these three fixed effects, but other than that it was identical to the full model.

In both models (*Model 2: carrot day* and *Model 3: with days since grape*), the continuous variables (including the log-transformed variable *time*) and the offset terms were z-transformed to make the model more likely to converge and to improve the interpretability of the estimates (Schielzeth 2010). Originally for both models, we started with a maximal model as suggested by Barr et al. (2013), which included correlations among random intercepts and random slopes (maximal model). Due to convergence issues and some of the correlations being estimated close to one (non-identifiable correlations), random correlations were excluded since this does not tend to increase the Type 1 error (Barr et al. 2013). Confidence intervals for the model estimates were obtained for all models by parametric bootstrapping (1000 replications). Furthermore, for all models, we determined the variation inflation factors (VIF) values to rule out collinearity. The VIF values in all the models were below 2, indicating that collinearity was not an issue (Maximum VIF values: *Model 2: carrot day*, 1.602; *Model 3: with days since grape*, 1.342). Marginal *R*^2^ values were calculated for all models to assess the percentage the fixed effects contributed to the variance in the response variable (Nakagawa and Schielzeth 2013).

## Results

**Fig. 2:**
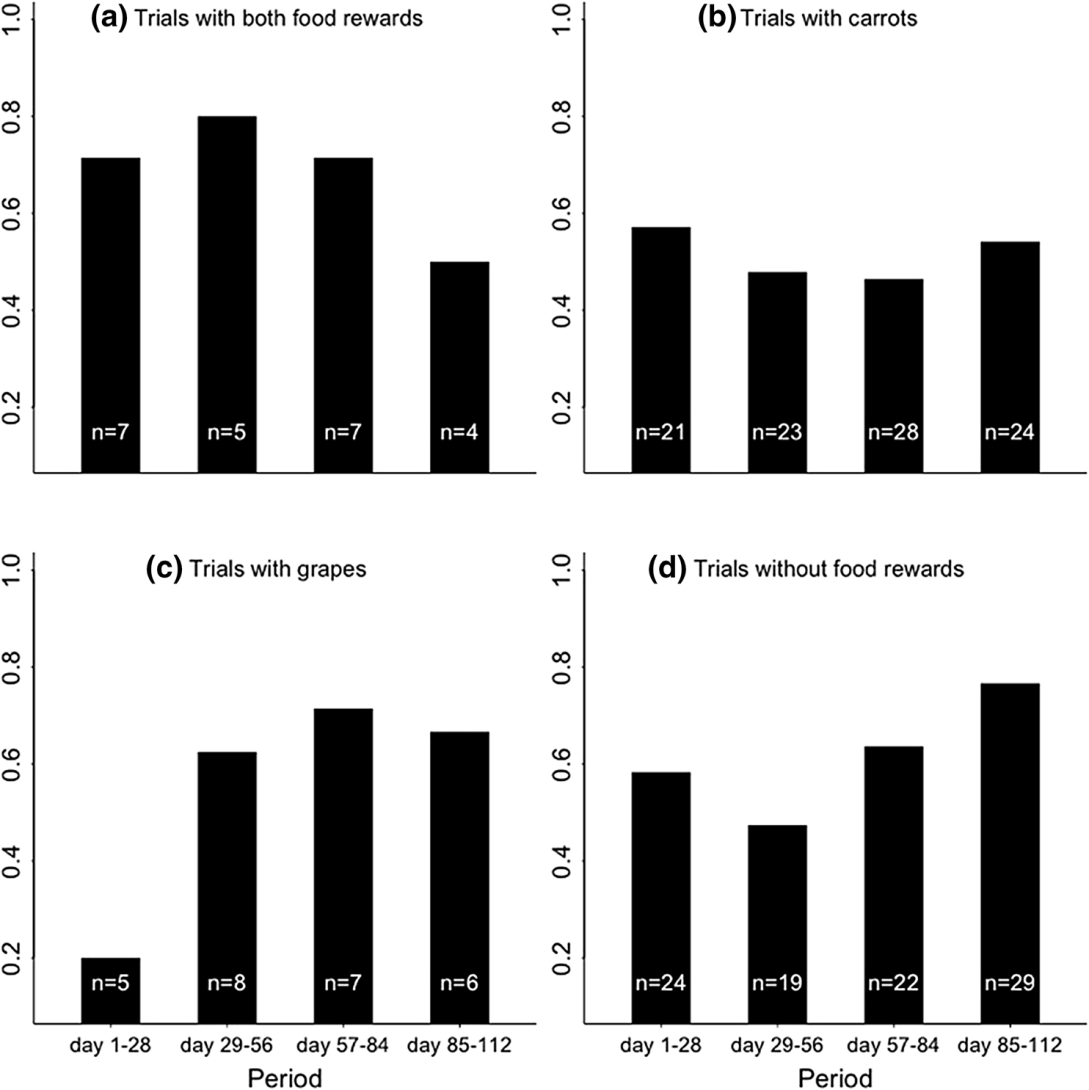
The proportion of searching at a grape location as a first choice during the course of the study (i.e. within the testing phase) for the four different types of day: **a** trials with both food rewards; **b** trials with carrots hidden; **c** trials with grapes hidden; and **d** trials without food rewards). The proportions were determined based on the number of first choices that were made during a 28-day period (i.e. approximately a quarter of the study period. We aggregated the data over this time period, due to the limited number of first choices on days with grapes and days with both food rewards)

During the 113 days of the testing phase, a total of 245 choices of location were recorded for a total of seven individuals. Throughout this period three subjects, (a juvenile mandrill, and an adult female with a newborn infant), did not make a first choice. Additionally, of the seven individuals, two adult females only once made it first to a food location. These individuals were not included in the statistical analysis for both the time interval models to increase model stability. This resulted in 243 choices (made by five individuals) throughout the study of which 23 first choices were made during trials with both food rewards, 96 during trials with carrots hidden, 26 during trials with grapes and 98 during trials without food rewards (Fig. 2). On average, individuals searched at 0.95 (SD = 1.20) unvisited locations during each trial (not including the abovementioned individuals that had made only one or no first choice; Table 4).

Throughout the study, there was one day with snowfall (day 53, a day without food rewards) during which none of the mandrills searched at one of the food locations during the trial. Furthermore, there were seven trials throughout the study during which not all six locations were searched at within the one hour of observation time (six of these trials were on days with no food rewards and one on a day with carrots).

Both *Model 2: carrot day* and *Model 3: with days since grape* were preferred over their respective null models (*Model 2: carrot day*: *χ*2 = 15.294, df = 6, *p* = 0.018; *Model 3: with days since grape*: *χ*2 = 11.835, df = 5, *p* = 0.037). The fixed effects structures of both models explained 15% of the variance in the response variable (Tables 1 and 3). We found a significant interactive effect of the type of day and the days since start on the probability that the first choice of a mandrill corresponded to a carrot location (*Model 2: carrot day*: Table 2). This interaction revealed that the mandrills were more likely to search first at a carrot location on carrot days, but only when more days since the start of the study had passed by and the monkeys had more trials to learn. It further revealed that the probability to go to a carrot location was lower on days when no food was hidden, but only at the later days of the study. Since the observations from the days with grapes were excluded from *Model 2: carrot day*, there are no results with regard to the choice of the mandrills on these days. The fixed effect of *cue grape* showed instability (Table 2). There was no significant effect of *cue carrot* on the first choice of individuals.

**Table 3:** Summary of results of binomial GLMM: effect of *carrot day* and *days since grape* on searching at a grape location as a first choice (*N* = 243)

	Original Estimate	Std. error	*P-*value	Estimate min.^a^	Estimate max.^a^	Lower CI^b^	Upper CI^b^
(Intercept)	1.099	0.329		0.868	1.415	0.568	1.883
Cue grape (absence)	− 0.167	0.392		− 0.319	− 0.126	− 1.147	0.489
Cue grape (presence)	0.100	0.580		− 1.055	0.517	− 1.301	1.213
Cue grape			0.870				
Cue carrot (absence)	− 0.853	0.500		− 1.198	− 0.253	− 1.855	0.264
Cue carrot (presence)	− 0.672	0.465		− 0.932	0.190	− 1.611	0.375
Cue carrot			0.241				
Days since grape	− 0.109	0.169	0.517	− 0.282	0.032	− 0.471	0.258
Days since start	0.670	0.214		0.633	0.032	0.260	1.230
Carrot day (Yes)	− 0.779	0.421		− 1.568	− 0.399	− 1.686	− 0.034
Days since start*Carrot day (Yes)	− 0.843	0.304	0.0373*	− 1.388	− 0.650	− 1.556	− 0.246

Full model vs. control model comparison: *χ*^2^ = 10.518, df = 4, *p* = 0.033, *N*_first choice grape_ = 142, *N*_individual_ = 5. Marginal *R*^2^ = 0.15

Significant *P*-values are marked with an asterisk

^a^ Minimum and maximum estimated coefficients derived by taking out the levels of each random effect one at a time

^b^ Bootstrapped 95% confidence interval

**Fig. 3:**
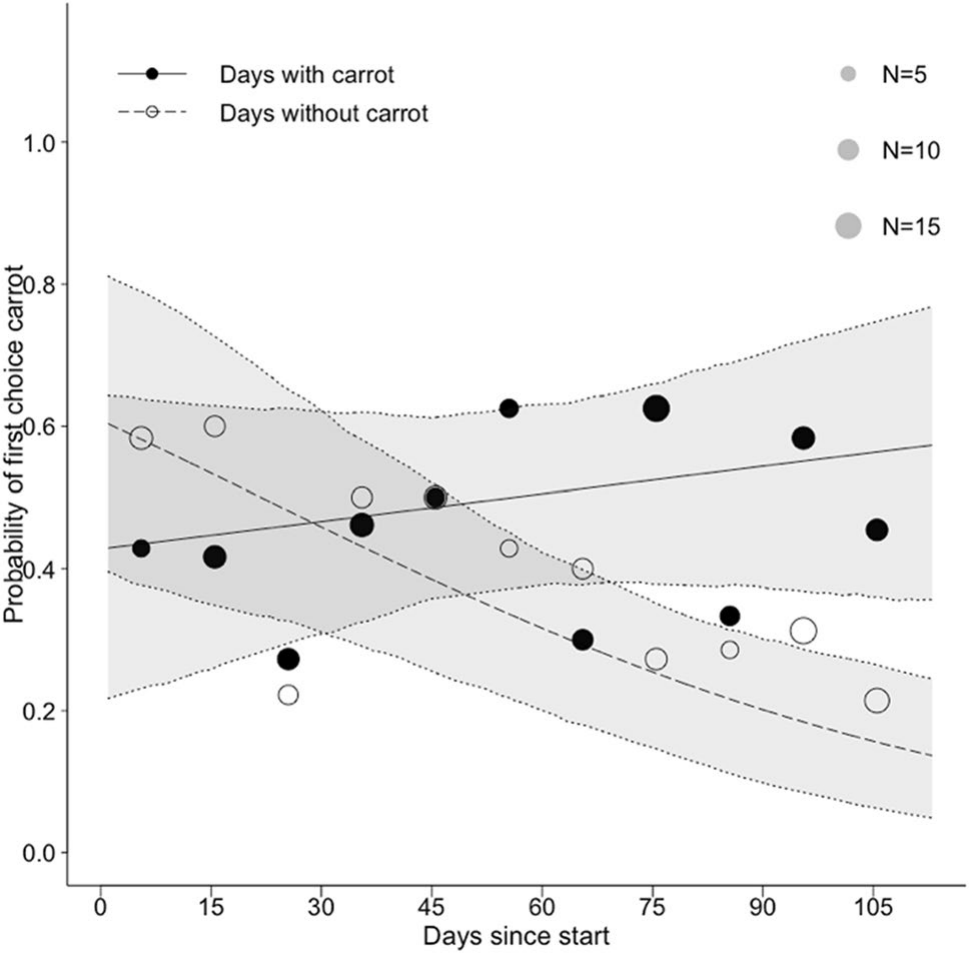
The probability of choosing a carrot location as a first choice during the course of the study (i.e. within the testing phase). The circles represent the proportion of first choice carrot locations in a 10-day period (i.e. the number of times a carrot location was chosen first divided by the total amount of first choices that were made during the 10-day period). The black circles represent the days with carrots hidden and white circles represent days without carrot. The size of the circles represents the number of first choices that were made during a 10-day period. The dotted line represents probabilities of choice predicted by the model for the days without carrot. The full line represents the probabilities of choice for the days with carrot. The shaded areas represent the bootstrapped 95% confidence interval

**Fig. 4:**
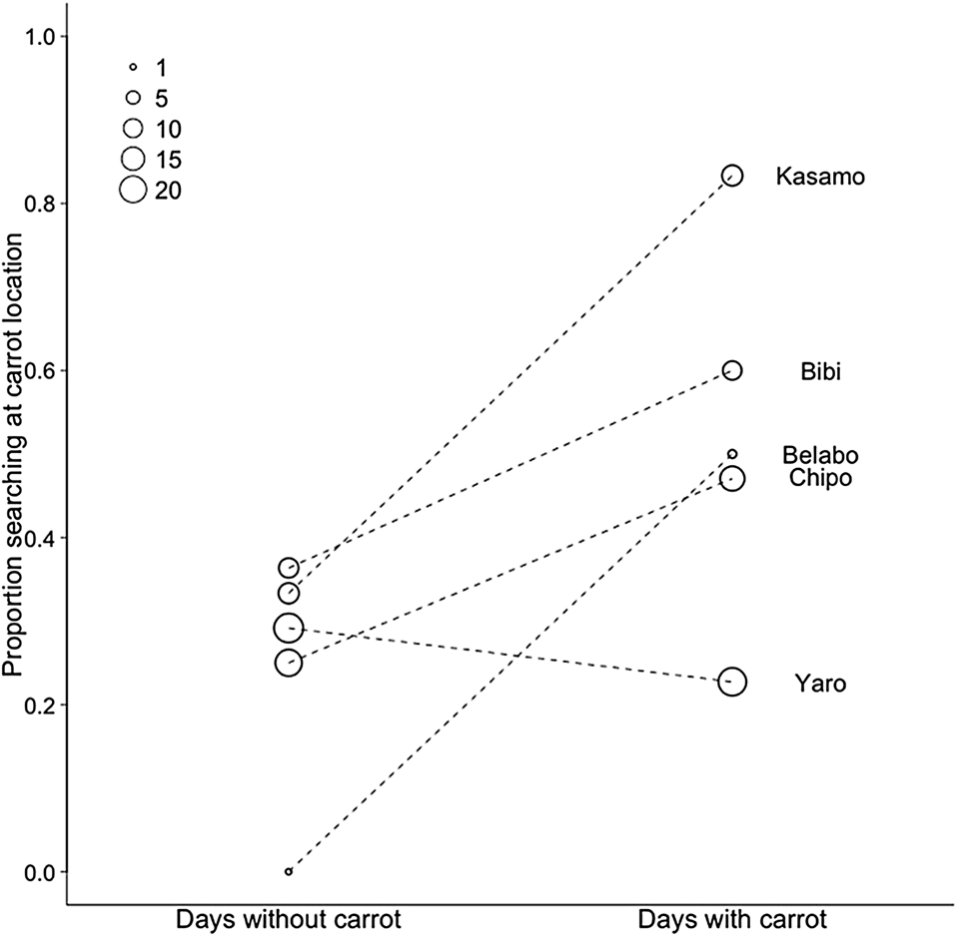
The proportion of searching at carrot locations as a first choice for individual mandrills during the second half of the study (day 56–113 of the testing phase). The circles represent the proportion of first choice carrot locations at days with carrot food rewards and days without carrot food rewards. The size of the circles represents the number of first choices that were made. The dotted lines indicate that the proportions for searching at a carrot location (on days with carrots and days without carrots) belong to the same individual. The names of the individuals are placed next to the circle that corresponded with their choices. Kasamo = adult male; Yaro = sub-adult male; Bibi, Belabo, Chipo = adult females

Moreover, in *Model 3: with days since grape*, there was also a significant interactive effect between *carrot day* and *day since start* on the probability that the first choice of a mandrill corresponded to a grape location (Table 3, Fig. 3). Similarly, this interaction revealed that during the course of the study, the mandrills were less likely to search first at a carrot location on days without carrot and more likely to search first at a carrot location on days with carrot (Fig. 4). In other words, similarly to model 2, we found that the probability to go to carrot increased on carrot day when more days since the start of the study had passed by. The fixed effects of *cue grape* and *cue carrot* showed instability (Table 3). There was no significant interactive effect of *days since grape* and *days since start* in the initial model and was, therefore, excluded from the model. The fixed effect *days since grape* showed instability in the final model, indicating the presence of influential cases (Table 3). These influential cases have the capacity to change the conclusion with regard to the effect of the *days since grape*, thereby preventing us to draw any conclusions with regard to this fixed effect.

## Discussion

With this study, we investigated whether captive mandrills were able to learn time intervals on the order of days. Our study provides evidence that captive mandrills are able to learn the two-day renewal intervals in a naturalistic foraging situation, while there is no evidence whether they can learn five-day renewal intervals. Thereby, the observations on mandrills in this study have provided evidence for the when component of the *where-when-what* memory in mandrills, i.e. the mandrills in this study needed to know when the carrots were last present to deduce when to visit carrot locations again. To our knowledge, this is the first study providing evidence that mandrills can keep track of the elapsed time since a specific event in the past (on the order of days).

### Temporal memory

Throughout the study, mandrills became more likely to first search at carrot locations on days with carrot, while they became less likely to search at carrot locations on days without carrot. These results indicate that captive mandrills were able to learn time intervals of two days and that they associated the two-day time interval with the carrot (locations). Furthermore, visual inspection of the probabilities of choosing a carrot location as a first choice (as predicted by *Model 3: with days since grape*), reveals that around day 30 of the testing phase the mandrills started to search (as a first choice) more often at carrot locations on carrot days in comparison to days without carrot (as revealed by the crossing point of the two lines in Fig. 3). Thereby, the results suggest that the mandrills in this study have learned the carrot time interval at approximately day 30 of the testing phase (or after 21 times of exposure to carrots and the associated interval).

Our initial model to test the temporal abilities of the mandrills was unstable with regards to the level of *days with grape* and *days with both rewards* (within the fixed effect of *type of day*) due to insufficient first choices by some individuals within these levels. Therefore, these data belonging to these levels were omitted from the data analysis. Hence, it is not possible to conclude whether the mandrills were able to learn the longer time interval of grape reoccurrence exactly. Furthermore, we additionally investigated whether mandrills had at least an imprecise notion of the five-day time interval as might be expected as a result of the scalar property of interval timing (i.e. the perception of time is more variable for longer time periods; Gibbon 1977; Shettleworth 2009). We expected that the mandrills would be more likely to go to grape locations with an increasing number of days that had passed since the last grape day. However, the fixed effect *days since grape* in this model showed instability. As a consequence, we were unable to demonstrate whether the mandrills had gained an imprecise comprehension of the five-day interval of grapes.

Throughout the study, the mandrills became more likely to go to carrot locations on carrot days, while on days without food rewards they became more likely to search first at grape locations (not searching at a carrot location corresponds to searching at a grape location; Table 2). Visual inspection of the proportion of grape location choices at different types of day during the course of the study also suggests that the mandrills became more likely to go to carrot locations on days with carrots and that they chose for grape locations first when there were no carrots (on days without food rewards and days with grapes; Fig. 2). If the mandrills would have learned the five-day interval of grapes, we would have expected them to search first at grape locations on days with both food rewards. However, this is not what we found. Additionally, throughout the study, the mandrills did not search more often at grape locations on days with both food rewards. Thus, the mandrills also did not seem to be improving over time with regards to the five-day time interval. This could be the result of a strategy that the mandrills used, in which they choose the foraging option that has the highest chance of foraging success (i.e. the food source that is present 50% of the days compared to 20% of the days) when they are uncertain about the longer time interval, but more certain about the smaller time interval. Such uncertainty about the longer time interval can be expected due to the scalar property of interval timing (Gibbon 1977; Shettleworth 2009).

Our results indicate that, throughout the study, the mandrills became more likely to first search at carrot locations on days with carrot. An alternative explanation for these findings could be the use of olfactory information to localize the carrots on carrot days. We consider this unlikely since the food sources were buried underground, thereby limiting olfactory cues. Furthermore, this alternative explanation is not supported by our results because one would expect the mandrills to learn to go to the correct location in several days rather than in 30 days if they were able to use olfactory cues, especially because the food sources were familiar to them at the start of the study.

The choices of the mandrills in the second half of the testing phase suggest that most mandrills that made regular first choices were able to learn the two-day interval (Fig. 4). However, the first choice of the sub-adult male falls mostly upon grape location regardless of the presence of carrots that day. This could reflect that instead of using memory the sub-adult male uses a different strategy in which it rapidly searches at more than one locations while having a preference for grapes (Table 4). This strategy could be especially usefull in the presence of a higher-ranking adult male in the group which could displace the sub-adult male and monopolize food.

**Table 4:** Average number of locations searched at during trials on different daytypes

	Age class	Sex	No food reward	Carrot day	Grape day	Both food rewards	Total number of trials searched for food
Belabo	A	F	0.13	0.07	–	–	7
Bibi	A	F	0.24	0.42	0.6	0.45	31
Chipo	A	F	1.22	1.58	1.10	1.00	83
Yaro	SA	M	2.27	2.09	2.40	2.09	102
Kasamo	A	M	0.82	0.63	0.60	0.82	46

The number of locations that was searched at, is based only on the instances that a search event at a location was not preceded by another individual searching for food at the same location within the same trial

*M* male, *F* female, *A* adult, *SA* subadult

Our findings that the mandrills were able to learn the shorter two-day time interval stand in contrast to the results of a study with rhesus monkeys (*Macaca mulatta*) that were tested in a different paradigm mimicking the perishability of food (Hampton et al. 2005). In the study by Hampton et al. (2005), rhesus monkeys were presented with a ‘perishable’ preferred food item and ‘unperishable’ less-preferred food item. With this design, the rhesus monkeys were unable to learn that, while both food items would be present after one hour, only the less-preferred food would be available after a longer time interval of 25 h.

The study by Hampton et al. (2005) and our study have used a different paradigm. This may explain why the results suggest that rhesus monkeys were unable to learn that the different time intervals are associated with the two types of food. The time-estimation in the study by Hampton et al. (2005) was in the range of hours, while our study had time intervals in the range of days and these different time scales could rely on different timing mechanisms (Ivry and Schlerf 2008). This difference in the set-up could underlie the differences in the performances of these two primate species. However, we propose an additional explanation. The rhesus monkeys were socially isolated during the testing, while the mandrills in our study were in a competitive group setting. In the first place, it may seem that the group setting did not contribute to the mandrills succesfully learning the two-day time interval, as the mandrills did not appear to be using social synchrony cues. However, a naturalistic foraging setting in which subjects forage in a social group can be beneficial for the learning and memory performances of the subjects due to a reduction in stress and increased motivation for reasons of direct competition (Janmaat 2019; Cronin et al. 2017; Menzel and Juno 1985). The finding that they did not appear to use social synchrony cues could have other reasons, which will be discussed below.

Although the paradigm is very different using different time intervals, it is interesting to speculate about the effects of the diet on potential genetic predispositions for learning these intervals. Mandrills are more reliant on fruit as a food source than rhesus monkeys (63% of the diet of rhesus monkeys is composed of fruit vs. 92% in mandrills) (DeCasien et al. 2017; Jones et al. 2009; Smith and Jungers 1997). The variability in fruit availability in tropical forests may induce long periods of fruit scarcity for primate species that are mainly reliant on ripe fruit (Janmaat et al. 2014; Chapman et al. 2005; Terborgh 1986). The capacity to remember elapsed time may provide primates with first access to ripe fruits and, therefore, ripe-fruit reliance may have formed a greater selective pressure for these time-estimating capacities in mandrills relative to rhesus monkeys. Currently, there are only a limited number of studies that have investigated the ability of primates to remember elapsed time. Evidence for the ability to remember time intervals longer than a day has only been found in two other primate species: chacma baboons and black capuchin monkeys, both of which have a high preference for fruit (Hill and Dunbar, 2002; Izar et al. 2012; Noser and Byrne 2015; Janson 2016; Tujage and Janson 2017). To identify if fruit reliance has formed a selective pressure on the time-estimating abilities in primates, it is necessary to study these abilities in more primate species with a paradigm that is suited for primates, such as the one used in our study. Information regarding the time-estimating abilities across a range of primate species can provide an opportunity to identify selective pressures by examining the relationship between this ability and ecological (such as the percentage of fruit in the diet) and/or social characteristics of primates (Janmaat 2019; Rosati 2017; Maclean et al. 2012; Harvey and Pagel 1991).

### Synchrony cues

The second aim of this study was to investigate whether captive mandrills used synchrony cues in a naturalistic foraging context. In both Both *Model 2: carrot day* and *Model 3: with days since grape*, the fixed effect *cue grape* was unstable; hence, we are not able to conclude anything regarding the ability of mandrills to use grape synchrony cues. The effect for *cue carrot* was stable but not significant. Even though the mandrills seemed to know the carrot locations as they were able to learn to go to carrots after two days had passed, they were not more likely to search at a carrot location after another individual had searched and found carrot. Thus, with this study design, we were unable to provide evidence that the mandrills were able to learn how to use synchrony cues provided by conspecifics within the duration of the study.

A lack of an effect of the cues could be due to the ephemerality of the event of a mandrill digging and finding (or not finding) food at one of the food locations. When mandrills found food at a carrot or grape location, they would often rapidly store the food in their cheek pouches and would then later retrieve it for consumption, hence, other conspecifics may not have seen the food. In the cases that a mandrill did not find food, it would often quickly go to another food location or it would just go away from that location. Thus, what conspecifics would see of these previously visited food locations could be limited to removed willow branches next to the food location and dug-up dirt in these locations. Thus in these cases, conspecifics would perceive a cue that another individual had searched at that food location but would have gained no information about the presence or absence of food at that location. Furthermore, since mandrills would often quickly go to food locations, they might have limited time to observe others and perceive the cues before they searched at one of the food locations. Therefore, the mandrills could have preferentially used their own temporal memory rather than synchrony cues as a strategy to localize food in this study. Another possibility is that the food sources were too close together. By the time that one individual had searched in one location, the others may have already exploited one of the remaining locations.

An alternative explanation is that the mandrills were selective in who they monitored. A study with captive mandrills by Schino and Sciarretta (2016) indicates that mandrills were more often monitoring high-ranking groupmates than low-ranking groupmates. Similarly, the mandrill group at Artis Royal Zoo could also be selective in who they monitor and due to this selective monitoring, it could be possible that they use cues provided only by high-ranking individuals. How social information and social monitoring affect foraging decisions in a food competition setting, is an intriguing topic for future research.

### General discussion

It has been suggested that temporal and spatial cognitive abilities may have provided an evolutionary advantage for large-brained primates that need to sustain the energetic costs of their large brains (Milton 1981; Janmaat et al. 2014; Trapanese et al. 2018). However, there is little information regarding the temporal cognitive capacities in primates and how these capacities vary between species. As such, our study provides valuable new information. To understand the evolutionary pressures that may have selected for larger brains, there is a need to improve our knowledge of the temporal cognitive capacities of primates. We encourage other scientists to use the adaptation of the procedure of Fuhrer and Gygax (2017) that we used in this study, to examine the temporal cognitive capacities in a naturalistic foraging context in a variety of primates in captivity in a highly comparative manner. This procedure of investigating mutliple different time intervals within the same study is beneficial in extracting as much information as possible from a group of naïve subjects (naïve with respect to this procedure). In addition, the two intervals we used resulted in four types of days which facilitate the interpretation of the results, especially when considering which potential food location the subjects visited on days when there was no food hidden there (i.e., on days that were not carrot days subjects searched less on carrot locations; cf Janmaat 2019). There are of course also limitations of this procedure, e.g. it could be difficult to provide evidence that an animal has learned the longer time interval, if it uses the strategy to choose the foraging option that has the highest chance of foraging success when it is uncertain (i.e. on days when both food rewards are present). Also, one would need separate groups of naïve subjects to test whether the measured effects of the time intervals is independent of the type of food offered.

Furthermore, our study has shown that cognitive enrichment with renewable hidden food sources could be implemented in the daily activity of zoo-housed mandrills and that they provide these animals with cognitive challenges that they could successfully cope with. This study illustrates how studies on cognitive performances can coincide with enrichment (Hopper et al. 2016). Dealing with cognitive challenges (that animals are able to successfully solve) has been suggested to be an important source of positive emotions in animals and has been suggested to lead to improved animal welfare (Meyer et al. 2010; Clark 2017). The adaptation of the procedure of Fuhrer and Gygax (2017) that we used throughout this study, could thus potentially be used in a variety of zoo animals as part of a cognitive enrichment program to further improve the welfare of zoo-housed animals. Additionally, our study provides an example of how cognitive enrichment can stimulate foraging behaviour that animals display in the wild (i.e. removal of plants and digging for an associated underground food source).

## Supplementary Information

Below is the link to the electronic supplementary material.

Supplementary file1 (DOCX 18 KB)

Supplementary file2 (MP4 75901 KB)

## Data Availability

The data used for this study are available from the corresponding author on request.

## Code availability

The code used in this study is available from the corresponding author on request.
