# Supplementary material for: Mandrills learn two-day time intervals in a naturalistic foraging situation
Source: Anim Cogn. 2020 Nov 30;24(3):569–82. doi: 10.1007/s10071-020-01451-7 (PMC8128742; doi:10.1007/s10071-020-01451-7)
Supplement: Supplementary file 1 — Supplementary file1 (DOCX 18 KB) [file 10071_2020_1451_MOESM1_ESM.docx]

**Supplementary Material for:**

# **Mandrills learn two-day time intervals in a naturalistic foraging situation.**

**Kavel C.D. Ozturk^1^ • Martijn Egas • Karline R.L. Janmaat^1,2,3^**

1. Department of Evolutionary and Population Biology, Institute of Biodiversity and Ecosystem Dynamics, University of Amsterdam, Science Park 904, 1098 XH Amsterdam, The Netherlands.

2. Department of Cognitive Psychology, Leiden University, Pieter de la Court, Wassenaarseweg 52, 2333 AK, Leiden, The Netherlands.

3. ARTIS Amsterdam Royal Zoo, Plantage Kerklaan 38-40, 1018 CZAmsterdam, The Netherlands.

Correspondence:

Kavel C.D. Ozturk, MSc,

Email: kavelcaglaozturk@gmail.com

**Table S1.** Summary of results of binomial GLMM with stability issues (Model 1): interactive effect of daytype (4 levels: no food day, carrot day, grape day, both carrot & grape day) and days since start on searching at a grape location as a first choice (N=237).

|  | **Original Estimate** | **Std. error** | ***P-*value** | **Estimate min.^a^** | **Estimate max.^a^** | **Lower**  **CI^b^** | **Upper**  **CI^b^** |
| --- | --- | --- | --- | --- | --- | --- | --- |
| (Intercept) | 0.119 | 0.318 |  | -0.191 | 0.502 | -0.489 | 0.769 |
| Cue grape (absence) | -0.009 | 0.427 |  | -0.291 | 0.108 | -0.987 | 0.678 |
| Cue grape (presence) | -0.330 | 0.741 |  | -1.922 | 0.788 | -3.115 | 1.027 |
| Cue grape |  |  | 0.903 |  |  |  |  |
| Cue carrot (absence) | -1.080 | 0.531 |  | -1.611 | -0.390 | -2.204 | 0.121 |
| Cue carrot (presence) | -0.698 | 0.478 |  | -0.959 | 0.026 | -1.590 | 0.121 |
| Cue carrot |  |  | 0.125 |  |  |  |  |
| Days since start | -0.113 | 0.246 |  | -0.446 | 0.100 | -0.666 | 0.442 |
| Type of day (grape & carrot) | 0.946 | 0.671 |  | -0.961 | 2.903 | -0.385 | 3.186 |
| Type of day (grape) | 1.111 | 0.809 |  | 0.766 | 1.320 | -0.364 | 3.812 |
| Type of day (no food) | 1.010 | 0.522 |  | 0.512 | 1.738 | 0.043 | 2.200 |
| Days since start:  Type of day (grape & carrot) | -0.182 | 0.586 |  | -1.113 | 0.064 | -1.792 | 1.103 |
| Days since start:  Type of day (grape) | 0.879 | 0.820 |  | 0.082 | 1.725 | -0.854 | 3.562 |
| Days since start:  Type of day (no food) | 0.821 | 0.343 |  | 0.681 | 1.366 | -0.854 | 3.562 |
| Days since start:  Type of day |  |  | 0.100 |  |  |  |  |
| Full model vs. control model comparison: χ^2^ = 14.266, df = 7, p = 0.0466, N_first choice grape_= 138. | | | | | | | |

^a^ minimum and maximum estimated coefficients derived by taking out the levels of each random effect one at a time

^b^ bootstrapped 95% confidence interval
